# Detection and characterization of spontaneous internal deletion mutants of *Beet Necrotic *yellow *vein virus *RNA3 from systemic host *Nicotiana benthamiana*

**DOI:** 10.1186/1743-422X-8-335

**Published:** 2011-07-01

**Authors:** Ying Wang, Huiyan Fan, Xian-Bing Wang, Min Li, Chenggui Han, Dawei Li, Jialin Yu

**Affiliations:** 1 State Key Laboratory for Agro-biotechnology and Ministry of Agriculture Key Laboratory for Plant Pathology, China Agricultural University, Beijing 100193, PR China

**Keywords:** Defective RNA, Beet Necrotic Yellow Vein virus(BNYVV), N protein

## Abstract

**Background:**

*Beet Necrotic Yellow Vein virus *(BNYVV) is a member of the genus *Benyvirus *causing a worldwide sugar beet disease rhizomania. BNYVV contains four or five plus-sense single stranded RNAs. In altered selective conditions, multipartite RNA viruses of plant are prone to undergoing internal deletions, thus turning into Defective RNAs (D RNAs). Although several D RNAs have been reported in BNYVV infection, the spontaneous internal deletion mutants responsible for severe symptom in systemic host *Nicotiana benthamiana (N. benthamiana*) are not described so far.

**Results:**

Systemic host *N. benthamiana *was inoculated by Chinese BNYVV isolates. RT-PCR and Northern blot showed that the D RNAs forms of BNYVV RNA3 were present in the systemic infection of the *N. benthamiana*. Three distinct D-RNA3s, named as D-RNA 3α, D-RNA 3β and D-RNA 3γ, were made into infectious clones. When inoculated on the *N. benthamiana*, the *in vitro *transcripts of D forms exhibited more stable than that of wild-type RNA3 in systemic movement. Among the detected mutant, the p25 protein frame-shift mutant (D-RNA3α) induced obvious necrotic lesions on *Tetragonia.expansa (T. expansa*) and pronounced systemic symptom on the *N. benthamiana*. The D-RNA3α was further mutated artificially to pre-terminate the downstream N protein, leading to the abolishment of the pathogenicity, indicating the N protein was responsible for the necrotic symptom.

**Conclusion:**

Our studies demonstrated the internal deletion mutants of BNYVV-RNA3 were spontaneously generated in the systemic infection on *N. benthamiana*. The internal deletions didn't affect the efficient replication of D-RNA3s, instead by improving the stability and pathogenicity of RNA3 in the systemic host *N. benthamiana*. Besides, our results also suggested the downstream N protein of RNA3, but not the upstream p25 protein, may play an important role in the systemic infection on *N. benthamiana*.

## Background

Defective RNAs (D RNAs), which have internal deletion of viral sequences, have been described for a variety of plant viruses [1,2]. Some D RNAs interfere with replication of the helper virus (called as defective interfering or DI RNAs) and affect symptom phenotypes, whereas others have little effect [1,3]. Previous reports have shown the generation of D RNAs or DI RNAs was a general biological process among multipartite RNA viruses of plants [1,4].

*Beet Necrotic Yellow Vein virus *(BNYVV), transmitted by zoospores of *Polymyxa betae*, is a member of the genus *Benyvirus *causing a worldwide sugar beet disease rhizomania [5]. BNYVV contains four or five plus-sense single-stranded RNAs designated as RNA 1 to RNA 5 in descending order of molecular size, individually packaged into rod-shaped virions [6]. RNA 1 and 2 encode "house-keeping" genes involve in replication, virion assembly, cell-to-cell movement, silencing suppression and vector transmission [7-10]. Only RNA1 and RNA 2 are sufficient for virus replication in local lesion host and virus vascular movement in the systemic host such as *Spinacea oleracea *or *N. benthamiana *[11-14]. RNA 3, RNA 4 and RNA 5 are functional in the natural infection process [15]. p25 encoded by RNA3 is required for induction of classical rhizomania symptom on natural hosts and local lesion phenotype on leaves of local hosts, while the downstream N protein could induce necrosis outside of the context of a BNYVV infection [16-18]. The core region from nt1033 to nt1257 of RNA3 is essential for the vascular movement of BNYVV in *Beta macrocarpa*[14]. RNA4 encoded p31 is associated with efficient vector transmission, virulence and RNA silencing suppression in roots [19,20]. Some BNYVV isolates contain RNA5 encoding a single 26-kDa protein that can also enhance symptom severity in a synergistic fashion with RNA3 [21-27].

D RNAs usually contain in-frame deletions within one or more genes and in some cases may be associated with pathogenicity of virus [2]. Several D RNAs have been described in BNYVV infections [28]. During serial mechanical passages, D RNA-2a and D RNA-2b are generated by in-frame deletions in the read-through region of the coat protein, and the mutant strains could not be transmitted by the fungal vector, indicating that the read-through product is necessary for vector transmission [28]. Deleted forms of BNYVV RNAs 3 and 4 have been detected in field isolates and induced by mechanical inoculation [29]. In some isolates, RNA 3 and 4 are prone to be eliminated spontaneously, whereas they are usually persistent with shorten forms in others isolates [29-32]. When the *in vitro *transcripts of RNA 3 and 4 are inoculated on *Chenopodium quinoa *leaves, they usually generate internal deletions spontaneously within only one or two mechanical passages [33]. Besides the naturally occurring D RNAs, a series of artificial deletion mutants derived from BNYVV RNAs have been constructed and characterized to investigate the function of each gene on symptom expression of BNYVV [17,34-36].

BNYVV can infect *N.benthamiana *systemically, causing severe or mild symptom [11]. Early reports show that RNA4 encoded p31 is associated with severe symptom such as curling and stunting in *N. benthamiana*, whereas RNA3 is non-related with these severe symptom [20]. Furthermore, RNA3 of Japanese isolate O11 is not stable in the systemic infection, and usually eliminated during virus propagation in *N. benthamiana*[20]. However, the reason why the RNA3 is unstable in the systemic movement still remains to be determined.

In this paper, the Chinese isolate BN345 (RNAs 1, 2, 3, 4 and 5) and BN3 (RNAs 1 2 and 3) were used to infect *N. benthamiana*[37]. Unlike the eliminated RNA3 of reported isolate O11, the generation of internal deletion RNA 3 mutants (defined as D-RNA3s) occurred with a high frequency in the systemic infected leaves. Most interestingly, the D-RNA3s could cause more severe symptom than full-length RNA3. Sequencing of RT-PCR showed the D-RNA3s included three distinct deleted forms. The inoculation of *in vitro *transcripts revealed that the D-RNA3s were very stable in the systemic movement, and induced the obvious necrotic symptom in the *N. benthamiana*, which provided the first evidence showing the stability of viral RNAs could be improved by spontaneous deletion under the selective pressure of systemic plants. Besides, the severe necrotic along the vein of systemic leaves induced by D-RNA3s confirmed N protein is responsible for these symptom. Finally, the possibilities of D-RNA3s causing severe symptom in *N. benthamiana *were discussed.

## Results

### Detection of the shorten form RNA3 in the BNYVV-infected *N. benthamiana*

BNYVV has been reported to infect *N.benthamiana *systemically, causing severe or mild symptom [11], which was mostly associated with the viral RNA4 encoded p31 protein [20]. The RNA3 of isolate O11 was found to be eliminated at an initial infection process or disappear spontaneously during virus propagation in *N. benthamiana *[20].

**Figure 1:**
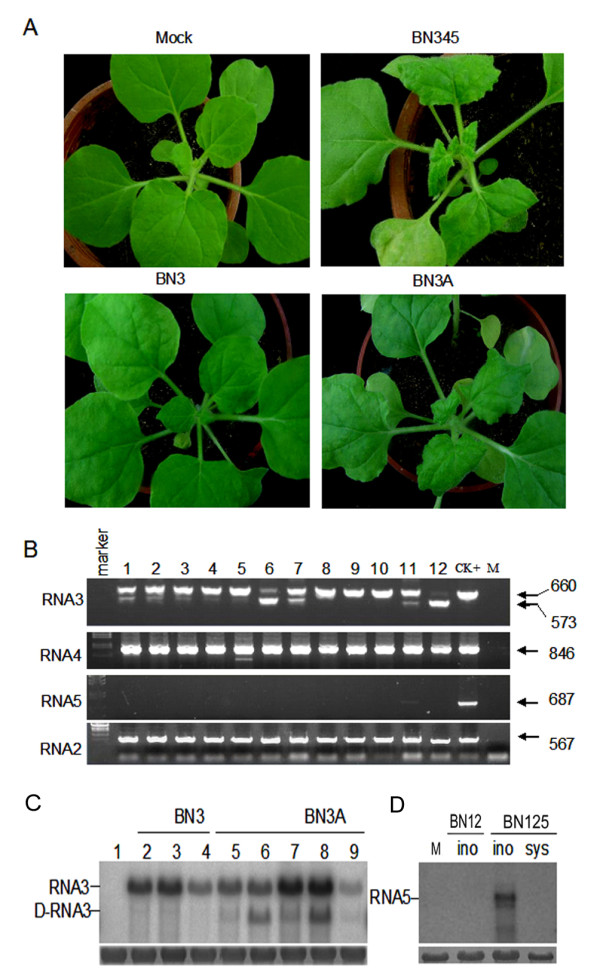
**The characteristic of viral RNA3, 4 and 5 in *N. benthamiana *infected by Chinese BNYVV isolates**. (A) The systemic symptom of isolates BN345, BN3 and BN3A on the *N. benthamiana *plants. The mock was inoculated by buffer. (B) The detection of RNA3, 4, 5 from the systemic leaves of the *N. benthamiana *plants by RT-PCR. Each lane represented an individual infected plant. The RNA from infected leaves of *T.expansa *was detected by RT-PCR as positive control (CK+ lane). The M (mock) was the result of RT-PCR from buffer infected *N. benthamiana *plants. The expected sizes of the PCR fragments were indicated on the right. (C) The northern detection of RNA3 and D-RNA3s from upper leaves of *N. benthamiana *individually infected by the isolate of BN3 and BN3A. (D) RNA5 detection by northern blot in the inoculated (ino) and systemic (sys) leave infected by BN12 or BN125.

To investigate the infection characteristic of RNA3 4 5 from a Chinese BN345 isolate (RNAs 1 2 3 4 and 5) in systemic host of *N. benthamiana*[37], we examined the viral RNA composition from the systemic infected leaves of *N. benthamiana*. The BN345 isolate could induce very strong symptom, such as dwarfing and curling, on the *N. benthamiana *at 14 day post inoculation (dpi) (Figure 1A). Consistent with the previous studies [20], the RNA4 could be detected in all of the 25 infected plants, and deletion forms RNA4 was present in one of infected plants, demonstrating that the high stability of RNA4 in the systemic infection of BNYVV(Figure 1B, RNA4 panel). In contrast, the RNA5 could be detected only in 2 out of 25 infected plants, suggesting the replication and movement of RNA5 was very low efficient in the BNYVV infected *N. benthamiana *(Figure 1B, and data not shown). To rule out the possibility that the co-infection of RNA3 and 4 inhibit the systemic movement of RNA5, the isolate only including RNA1 2 5 was inoculated on the *N. benthamiana*, showing the RNA5 could be detected only in the inoculated leave but not in the systemic leaves (Figure 1D). Thus, the low efficiency of RNA5 in the systemic infection was independent of the co-infection of RNA3 and 4. The detection of RNA3 showed intriguing results, in which 10 out of 25 infected plants only contained wild- type RNA3 (Figure 1B, lane 8, 9, 10 and data not shown), whereas the left 15 infected plants had both wild type RNA3 and some shorten forms RNA3 (Figure 1B, lane 1 to 7, lane 11 and data not shown). This results indicate that the RNA3, unlike the high stable RNA4 or defective RNA5, could keep the systemic infection activity but with high frequency of deletion forms in *N. benthamiana*.

As a control, the inoculation of local-lesion host *T. expansa *could support the efficient replication of the wild-type RNA3, 4, 5(Figure 1B CK+), indicating the stability of genomic RNA was affected by the different host.

### Isolation of the spontaneous internal deletion mutants of RNA3

To determine the function of D-RNA3s on the pathogenicity of BNYVV, the *N. benthamiana *were inoculated by BN3 isolate (including RNAs 1 2 and 3) [37]. Among the 8 infected plants, 5 plants developed strong curling in the upper leaves(Figure 1A, BN3A), while the other 3 showed no difference with plants infected by mock buffer (Figure 1A, BN3). Northern blot showed the BN3 contained dominant amount of full-length RNA3 and trace amount of D-RNA3s, while the BN3A comprised the full-length RNA3 and D-RNAs both in considerable amount (Figure 1C), implying the emerging D forms might be related with the severe symptom.

**Figure 2:**
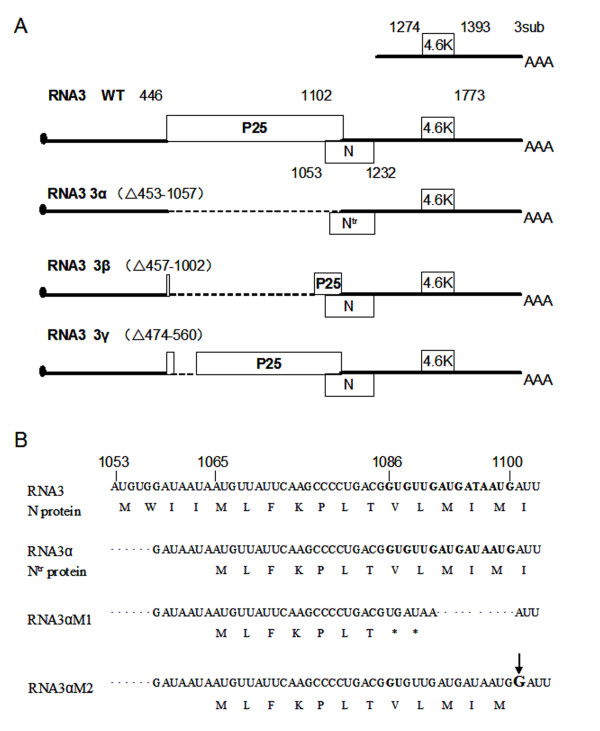
**The schematic structure of wild-type RNA3 and Defective RNA3 (D-RNA3s)**. (A) The schematic structure of wild-type RNA3, D-RNA 3α, D-RNA 3β and D-RNA 3γ. The deleted regions were indicated in nucleotides, and shown by broken lines in the structures. (B) The point (underline) and deleted mutants (broken line) were introduced into the D-RNA3α, leading to the pre-termination of N^tr ^protein, termed as D-RNA3αM1. The second mutant D-RNA3αM2 was constructed by inserting a G(arrow) into the 110nt of RNA3, resulted into a frame-shift mutant.

To profile the D-RNA3s generated in the systemic infection, we next sequenced the products of RT-PCR, showing that D-RNA3s contain three distinct internal deletions, respectively termed as D-RNA 3α, D-RNA 3β and D-RNA 3γ (Figure 2A). The analysis of sequence showed the deletion region in RNA 3α was from nt 453 to nt1057, causing a frame-shift mutation and abolishing the expression of p25 protein (Figure 2A). We noted that the first AUG of open reading frame (ORF) N was eliminated in the deleted region, therefore the translation of N protein might initiate by the second in-frame AUG, leading to generate the 5'-truncated product of N protein, referred as N^tr ^protein (Figure 2A and 2B). The deletion regions of D-RNA 3β and D-RNA 3γ were respectively located in the nt457-1002 and nt474-560, resulting in the deletion of 182 and 29 amino acid residues in the internal region of p25 protein, respectively (Figure 2A).

### Distinct symptom induced by D-RNA3s on the local hosts

**Figure 3:**
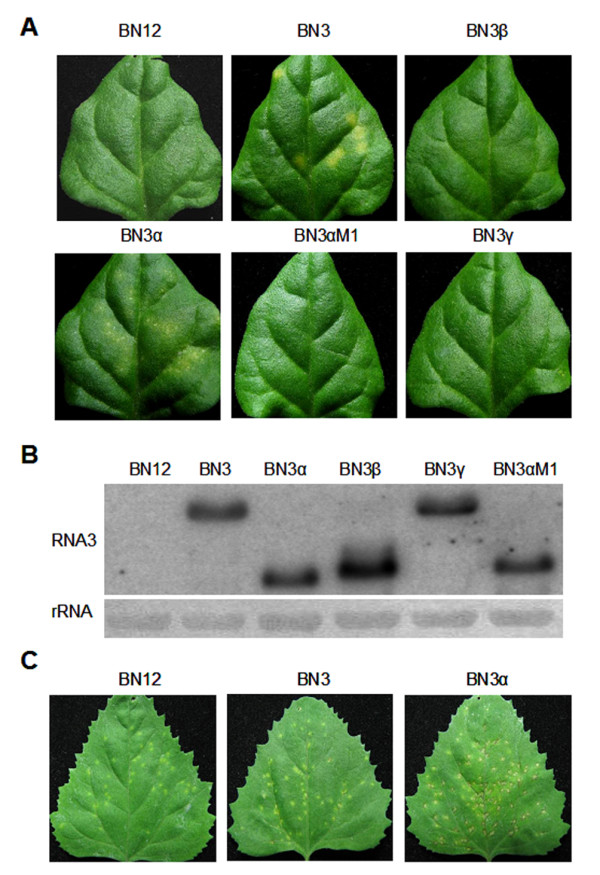
**Effects of BNYVV RNA3 and D-RNA3 mutants on the symptom of local lesion host**. (A) Symptom induced by RNA1, 2, alone or combined with in vitro transcripts of RNA3 and D-RNA3 on the inoculated leaves of *T.expansa *in 5 dpi. (B) Northern analysis of wild type RNA3 and D-RNA3 mutants from inoculated leaves of *T.expansa*. (C) Symptom induced by RNA1 2, alone or combined with in vitro transcripts of RNA3 and D-RNA3α on the inoculated leaves of *C. amaranticolor *in 5 dpi.

To further investigate the function of D-RNA3s, the full-length cDNA clones of the three D-RNA3s were constructed and inoculated on the local host. The classical local host *T. expansa *of BNYVV could be induced to form faint chlorotic spots by RNA1 and 2 (isolate BN12, Figure 3A). In contrast, the co-infection of full-length RNA3 and BN12 would cause yellow spots in 5 dpi, as described previously (Figure 3A) [17]. The D-RNA 3β or D-RNA 3γ, as did BN12 alone, only caused faint chlorotic spots, demonstrating the deletion form of p25 protein was deficient for inducing yellow spots. Surprisingly, the D-RNA3α induced obvious necrotic spots on *T. expansa *in 3 dpi, and then some yellow halos were shown around the necrotic spot in the few days later (Figure 3A). In the *C. amaranticolor*, the D-RNA3α could also induce necrotic spot in the inoculated leaves (Figure 3C). The northern results showed the D-RNA3s form could be replicated as efficiently as wild type RNA3, indicating the deletion region had no effect on the replication of RNA3 (Figure 3B).

To confirm the function of N^tr ^protein in the D-RNA3α, one mutant of D-RNA3α was constructed to pre-terminate the N^tr ^protein. The GUG and UUG codons near the beginning of ORF N^tr ^were converted to the UGA and UAA termination codons (Figure 2B). Meanwhile, the downstream sequence AUGAUAAUG (contain two initiation codons) was deleted to produce the mutant D-RNA3αM1 (Figure 2B). The D-RNA3αM1 could not induce necrotic spots when inoculated with RNA1 and 2 (Figure 3A), indicating the N^tr ^protein was the factor associated with the necrotic spots.

### Effect of D-RNA3 on the systemic infection of BNYVV

**Figure 4:**
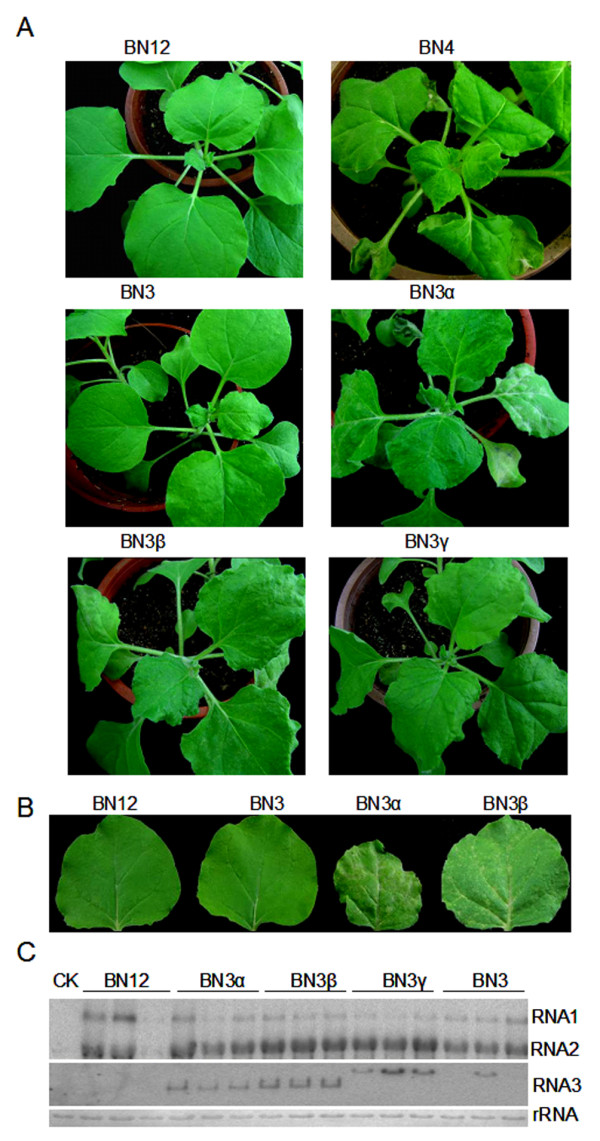
**Effects of RNA3 and D-RNA3 on systemic symptom and viral RNA accumulation in *N. benthamiana***. Symptom induced by RNA1, 2, combined with in vitro transcripts of RNA4, RNA3 or D-RNA3 on the *N. benthamiana *plants (A) and systemic leaves (B) in 14 dpi. (C) The Northern analysis of RNA1, 2 and RNA3 from the systemic leaves infected by RNA1, 2 or combined with wild type RNA3, as well as D-RNA3. Three individual infected plants were shown in each lane.

We next examined the effect of full-length and D-RNA3s on symptom expression on *N. benthamiana *by foliar rub-inoculation. BN4 including RNA1 2 and 4 induced obvious downward curling of the upper leaves, and the stunting of the infected plants as previous reported (Figure 4A) [20]. In contrast, BN12 alone or with full-length RNA3 only induced mild symptom on the *N. benthamiana *(Figure 4A). However, BN12 with D-RNA 3α caused severe necrotic spots in the systemic leaves, and distorted infected plant (Figure 4A and 4B). The symptom severities of plants infected with D-RNA 3β or D-RNA 3γ were milder symptom than RNA 3α, and some of the upper leaves had necrotic spots (Figure 4A and 4B). Together with the symptom of local host, these results suggested that the D-RNA 3α, wherein the full length of p25 protein was inactivated, remarkably enhanced the pathogenicity of RNA3.

**Figure 5:**
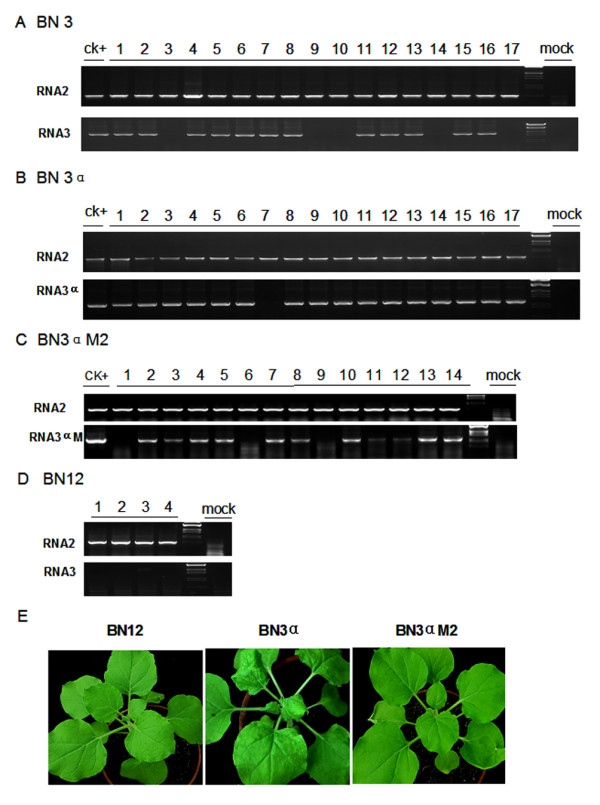
**The detection of RNA3, RNA3α and RNA3αM2 in the systemic infected plants**. The in-vitro transcripts of RNA3(A), RNA3α(B) and RNA3αM2 (C) were inoculated on the *N. benthamiana *plants respectively with total RNA including RNA1 and RNA2. In 14 dpi, the total RNA was extracted from systemic leave of each infected plant, and carried out for RT-PCR with the RNA2 or RNA3 specific primers. In each infected plant, the RNA2 detection as a positive control for successful infection. The plants infected by RNA1 2 were as negative control of RNA3 detection. (E) The symptom of infected *N. benthamiana *plants by BN12, RNA3α and RNA3αM2 in 15 dpi.

The northern blot of RNA from infected systemic leave showed the D-RNA 3α, D-RNA 3β and D-RNA 3γ existed in all the three detected plants, whereas only in one sample of the three detected plants, the full-length RNA3 could be detected (Figure 4C), demonstrating the full-length RNA3 might not be as stable as the short form deletion mutants. We also performed RT-PCR to show that the full-length RNA3 was detected in the 12 plants among the total 17 systemic infected *N. benthamiana *(Figure 5A). In contrast, the D-RNA 3α could be detected in 16 systemic leave among the total 17 infected plants (Figure 5). Thus, it is likely that the D-RNA 3α was more stable than wild-type RNA3.

To investigate the function of N^tr ^on the *N. benthamiana*, the D-RNA3αM1 mutant also was inoculated on the *N. benthamiana*. The RT-PCR results showed the D-RNA3αM1 could not infected *N. benthamiana *systemically (data not shown). Because the mutated region in the D-RNA3αM1 was coupled with essential factor of the systemic movement of RNA3 [14], the second mutant D-RNA3αM2 was constructed by inserting a G in the position of nt 1100 (Figure 2B). The D-RNA3αM2 could infect systemically on the *N. benthamiana *with lower efficiency than D-RNA3α, but not induce the necrotic lesion as the D-RNA3α (Figure 5C and 5E). Thus, we conclude that the N protein is not only required for induction of necrotic symptom, but also improved the efficiency of RNA3 systemic infection on the *N. benthamiana*.

## Discussion

For the multipartite RNA viruses, how the distinct functions of each RNA genome and protein comparatively facilitate the viral infection in different hosts and environment is an intriguing topic in the current studies. For accommodating to different conditions, the viruses usually prefer to replicate some necessary RNA, and discard the full-length or some part of other inessential genome RNA. For example, the RNA3 of isolate O11 was usually eliminated spontaneously in the *N. benthamiana *plants [20], and the RNA5 does not persist in many natural isolates [5,8]. Here, we examined the infection activity of the BNYVV RNA1, 2, 3, 4, 5 from Chinese isolate, and found the RNA1, 2, 4 successfully infected in the systemic leaves of host *N. benthamiana *as reported previously [20]. The RNA5 was almost under detected in the systemic leave whether co-infected with RNA1 2 3 4 or only with RNA1 2 (Figure 1), indicating the RNA5 was inessential factor at least on the *N. benthamiana*, although the RNA5 could enhance the pathogenicity of BNYVV in some other hosts [23,37]. At present, it is unknown whether the 5' and 3' un-translated region (UTR) or the open reading frame (ORF) affects the infection of RNA5 on the *N. benthamiana*. Different with instability of RNA3 from isolate O11 reported [20], the RNA3 of Chinese isolate generated some internal deletion forms in most systemic leaves of infected *N. benthamiana*. RNA3, 4, 5 could replicate with high efficiency in the local host *T. expansa*, indicating the different stability of RNA3, 4, 5 in systemic host is resulted from the distinct systemic movement activity, rather than the replication efficiency.

The full-length RNA3 was eliminated spontaneously in some systemic leaves of *N. benthamiana*, whereas the D-RNA3αwas more stable in the systemic movement (Figure 5). This paper provides an evidence to prove RNA3 could increase the systemic movement activity by spontaneously deletion on the *N.benthamiana*. Indeed, the northern results about the RNA3 of isolate O11 also contain some deletion forms in the previous results [20]. In addition to enhance the stability of RNA3 by the internal deletion, the inoculation assay in this study also demonstrated that one of D-RNA3s not only caused obvious necrotic spots on *T. expansa*, but also induced severe necrotic in the systemic leave of *N. benthamiana*. Therefore, the natural D-RNA3s, rather than full-length RNA3, acts as a new pathogenicity factor involving into the infection on the *N. benthamiana*, in addition to the reported RNA4 [20].

The sequence analysis of the natural D-RNA3α showed the deletion region (nt453-1057) led to a frame-shift mutation and abolished the synthesis of p25 protein (Figure 2A). In the genomic RNA3, the N protein with overlapping region with C terminal of p25 protein was confirmed to elicit a necrotic response through artificial cloning or expression by *Cauliflower mosaic virus*[17]. Here, the first four amino acids of N protein were deleted in the natural D-RNA3α. According to the previous results, the deletion of upstream sequence might activate the expression of N protein [17], which would be responsible for the necrotic symptom. To confirm this prediction, the N^tr ^protein pre-terminated mutants were constructed and inoculated on the local host and systemic host. The mutant of D-RNA3αM1 did not elicit the necrotic response any more in the local host as the D-RNA3α, indicating the N^tr ^protein was related with necrotic symptom. However, when inoculated on the systemic host *N. benthamiana*, the mutant of D-RNA3αM1, unlike the stable D-RNA3α, was unable to infect *N. benthamiana *systemically, confirming that the deletion region in the D-RNA3αM1 was also related with systemic movement as reported previously [14].

For confirming the function of N^tr ^protein in the systemic infection, the second mutant D-RNA3αM2 was constructed and successfully infected the *N. benthamiana *systemically without inducing the necrotic response. The results showed here indicated that the N protein, acting as inducer of necrotic symptom, was under-expressed in the wild-type RNA3 probably due to inhibition by the expression of p25. Based on these finding, we proposed that when the N protein was required for inducing symptom or enhancing stability in *N. benthamiana*, the wild-type RNA3 might adopt spontaneous deletion to improve the expression of N protein by sacrificing the temporarily non-functional p25 (Figure 5).

The previous results have reported various D-RNA3s from different hosts [8,33,35]. However, the natural D-RNA3s from systemic host *N. benthamiana *firstly reported in this paper was different with the previous sequences. Extensive experiments will be needed to determine the similarities and divergence of the natural deletions undergoing in different hosts, as well as the meaning of their emergence.

## Conclusion

These founding reported the spontaneous generation of internal deletion derived from BNYVV-RNA3 on *N. benthamiana*. The D-RNA3s were more efficient than full-length RNA3 during the systemic infection, and caused severe necrotic symptom on *N. benthamiana*. Results presented here provided evidences for understanding the evolution of viral genome RNAs under altered selective conditions in different hosts.

## Methods

### Plants and virus isolates

*T. expansa, N. benthamiana*, and C. *amaranticolor *were grown at 24 ± 1 °C under a 16 h light and 8 h dark regimen. In this studies, the BN12(RNAs 1+2), BN3(RNAs 1+2+3), BN345 (RNAs 1+2+3+4+5) were respectively derived from isolates BNYVV-Hu0, Hu3, and Hu as described previously [37]. BN4 (RNA1+2+4), BN3α (RNA1+2+3α), BN3αM (RNA1+2+3M1 or M2), BN125 (RNA1+2+5) were the mixture of total RNA from the inoculated leaves of *T. expansa *by BN12 and *in vitro *transcripts of each genome.

### Construction of full-length and defective RNA3 infectious cDNAs

The full-length and the defective RNA3s cDNA clones were amplified by RT-PCR from total RNA of isolate BN3 and BN3A. The forward primer 5'- CG*GAATTC*TAATACGACTCACTATAGAAATTCAAAA TTTACCATTA - 3' (*Eco*RI site in italics and T7 promoter sequence underlined) and reverse primer 5'-CC*TCTAGA *T _(26) _GTCAATACACTGACAGAGAA -3' [*Xba*I site in italics and oligo (dT) tract shown by T _(26)_] were used. The PCR products were purified and cloned into the pMD19-T vector to obtain a full-length cDNA clone named pMDR3 and three D RNAs cDNA clones, pMDRNA3α, pMDRNA3β and pMDRNA3γ (Figure 2A). Mutant RNA3αM (Figure 2A and 2B.) was produced by PCR-based, oligonucleotide directed mutagenesis within the pMDRNA3α clone. All constructions were sequenced and then used for transcription.

### In vitro transcription and inoculation

Infectious plasmids were linearized by *Xba*I and used by run-off transcription at 37°C for 2 hr with a T7 RNA polymerase kit as described by the manufacturer (Promega). The freshly synthesized transcriptions were mixed with total RNA of *T. expansa *infected by isolate BN12, the mixture supplemented with an equal volume of inoculation buffer (50 mM glycine, 30 mM K2HPO4, 1% bentonite, 1% celite, pH 9.2) were rubbed onto *T. expansa*, C. *amaranticolor *or *N. benthamiana *leaves. Local lesions generally appeared at 5-8 days post inoculation, while systemic symptom of *N. benthamiana *appeared at 12-14 dpi.

### RNA extraction and detection

Inoculated *T. expansa *leaves and systemic infected *N.benthamiana *were harvested at 7 and 14 dpi, respectively. Total RNA was extracted for RT-PCR detection and Northern hybridization, as described previously [38]. Probes were appropriate 32^P-^labeled cDNA specific for RNA1 (nt5815-6531), RNA2 (nt145-714), and RNA3 (nt445-1102) sequences, respectively.

The following primers were used for RT-PCR: for detection of RNA 2, the forward primer BN81 (5'-CGATGTCGAGTGAAGGTAGATA-3', nt145 to 164) and the reverse primer BN80 (5'- CTATTGTCCGGGTGGACTGG -3', complementary to nt962 to 712), for detection of RNA3, the forward primer BN78 (5'-GTGATATATTAGGCGCAGTTTATG-3', nt450 to 473) and the reverse primer BN77 (5'-TCATTATCATCAACACCGTCAG-3', complementary to nt1080 to 1101), for detection of RNA 4, the forward primer BN209 (5'-CTGATGGAGAGATATG-3', nt384 to 339) and the reverse primer BN210 (5'-CTAATCGTGATAAAAGACAAACCA-3'complementary to nt1205 to 1228) and for detection of RNA 5, the forward primer BN225 (5'-GATGGATATTGATCATTGTATG -3', nt459 to 480) and the reverse primer BN226 (5'-TCCACAATCATTATCATGAT-3', complementary to nt1124 to 1143).

## Competing interests

The authors declare that they have no competing interests.

## Authors' contributions

YW carried out most of the experiments and wrote the manuscript. HF anticipated the inoculation and the construction of D form RNA3s. XW and ML provided useful advice. CH, DL and JY conceived of the study and participated in its design and coordination. All authors read and approved the final manuscript.
